# Is ecology different when studied with citizen scientists? A bibliometric analysis

**DOI:** 10.1002/ece3.10488

**Published:** 2023-09-19

**Authors:** Bastien Castagneyrol, Baptiste Bedessem, Romain Julliard

**Affiliations:** ^1^ Université de Bordeaux, INRAE, BIOGECO Cestas France; ^2^ INRAE, LISIS Noisy‐le‐Grand France; ^3^ Centre d'écologie et des sciences de la conservation (UMR7204 MNHN, CNRS, SU) Paris France

**Keywords:** bibliometrics, citizen science, ecology, thematic map

## Abstract

Ecology is broad and relies on several complementary approaches to study the mechanisms driving the distribution and abundance of organisms and their interactions. One of them is citizen science (CitSci), the co‐production of scientific data and knowledge by nonprofessional scientists, in collaboration with, or under the direction of, professional scientists. CitSci has bloomed in the scientific literature over the last decade and its popularity continues to increase, but its qualitative contribution to the development of academic knowledge remains understudied. We used a bibliometric analysis to study whether the epistemic content of CitSci‐based articles is different from traditional, non‐CitSci ones within the field of ecology. We analyzed keywords and abstracts of articles published in ecology over the last decade, disentangling CitSci articles (those explicitly referring to citizen science) and non‐CitSci articles. Keyword co‐occurrence and thematic map analyses first revealed that CitSci and non‐CitSci articles broadly focused on biodiversity, conservation, and climate change. However, CitSci articles did so in a more descriptive way than non‐CitSci articles, which were more likely to address mechanisms. Conservation biology and its links with socio‐ecosystems and ecosystem services was a central theme in the CitSci corpus, much less in the non‐CitSci corpus. The situation was opposite for climate change and its consequences on species distribution and adaptation, which was a central theme in the non‐CitSci corpus only. We only revealed subtle differences in the relative importance of particular themes and in the way these themes are tackled in CitSci and non‐CitSci articles, thus indicating that citizen science is well integrated in the main, classical research themes of ecology.

## Graphical abstract

Citizen science is the production of scientific knowledge by professional scientists aided by people that are nonprofessional scientists (or the other way round). We used a bibliometric analysis to explore whether this type of collaboration creates peculiar knowledge.

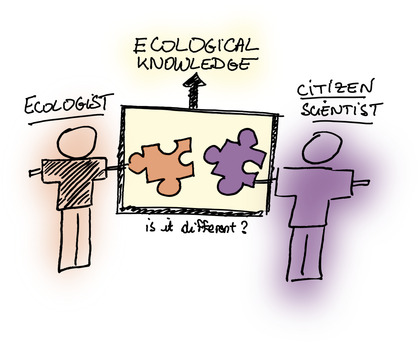

## 1 INTRODUCTION

Ecology roots in life science and natural history. The term “ecology” was first coined by Ernst Haeckel (Egerton, 2013) in 1866 to describe “the whole science of the relations of the organism to the environment including, in the broad sense, all the ‘conditions of existence’” (Haeckel, 1866, cited by Egerton, 2013). Ecology developed as an independent research area at the beginning of the XXth century and over the last 150 years, its definition evolved. Modern textbooks define ecology as “the scientific study of the distribution and abundance of organisms and the interactions that determine distribution and abundance” (Begon et al., 2006). This broad definition encompasses every living organism, from microbes to trees and the largest vertebrates, and every level of organization, from genes to the biosphere as a whole. To study living organisms and their interactions ecologists rely on observational, experimental, and modeling approaches. Their task is huge, financial resources are limited, and in the unprecedented context of the Anthropocene, advancing ecological knowledge has never been more urgent.

Citizen science—generally defined as the co‐production of scientific data and knowledge by individuals or groups of individuals that are not professional scientists in collaboration with or under the direction of professional scientists and scientific institutions (Eitzel et al., 2017)—appears in this context as a win‐win enterprise (Turrini et al., 2018) that benefits both science and society. Several studies addressed the impact of ecological citizen science (*CitSci*, henceforth) on people's knowledge and attitudes toward the environment (Aristeidou & Herodotou, 2020; Peter et al., 2019), but little is known on the impact of CitSci on the development of academic ecological knowledge.

Ecological research has much to gain from being conducted with nonprofessional scientists through CitSci (Couvet et al., 2008; Roger et al., 2019). For instance, a major challenge of ecology is the spatial and temporal replication of the results obtained at small spatial scale, and their generalization to larger scale (Filazzola & Cahill, 2021). Where there are people living, there is the potential for CitSci, even in the absence of professional ecologists. Citizen scientists and their observations, being or not guided by scientific protocols (Callaghan et al., 2021), largely increase the capacity of ecologists to characterize the “distribution and abundance of organisms” (Begon et al., 2006). Biodiversity maps and atlases are largely based on crowdsourced CitSci (Isaac et al., 2014; Turrini et al., 2018). For instance, global biodiversity databases such as iNaturalist (https://www.inaturalist.org/) or GBIF (https://www.gbif.org/fr/) are increasingly used by scientists as a relevant source of information for the study of local adaptation (Farquhar et al., 2023), the distribution and phenology of species (Forti et al., 2022) or the spread of invasive species (Rosa et al., 2022; Tran et al., 2022). Individuals and communities also have empirical and experiential knowledge that can advance academic knowledge in taxonomy or natural history, particularly in habitats that have been understudied such as Arctic areas (Danielsen et al., 2021). Among others, Tengö et al. (2021) insist on the role of indigenous and local knowledge to support citizen science practice (for instance, for water monitoring in Arnhem Land, Australia). Generally speaking, CitSci proves to be highly valuable in conservation biology (McKinley et al., 2017), but whether it can be of primary importance for ecology in general is questionable.

Ecology is a quantitative discipline. Many fields of ecology rely on sensors (e.g., eco‐physiology and eco‐acoustic) or highly specialized and onerous technologies (e.g., chemical ecology, molecular ecology, “omics” technologies) that are even not accessible to some research groups. Such parts of ecological research can hardly been addressed through CitSci (Pocock et al., 2014), unless it is developed as hybrid CitSci where volunteers are mostly involved in the collection of samples that are sent to professional scientists who keep the responsibility for further processing (Osenga et al., 2021). Likewise, although CitSci is of particular utility in urban environments where it allows getting into people's backyards, there are large parts of the world that are not accessible to citizen scientists; typically oceans, deep forests, deserts, and mountains. Consequently, specific fields of ecology such as marine or alpine ecology are likely less prone to rely on CitSci. Finally, because CitSci primarily relies on volunteer participation, some topics may trigger a greater interest than others. There are several CitSci initiatives targeting emblematic taxa such as birds (Jiguet et al., 2012), butterflies (Cima et al., 2020), or large vertebrates (Andrianandrasana et al., 2005), but much less about fungi or ground dwellers such as carabids or myriapods (but see Billaud et al., 2021; Sanden et al., 2021). If only certain research questions, methodologies, habitats, or taxa are relevant to CitSci, its contribution to ecology as a whole may be limited.

Bibliometrics is the quantitative analysis of research outputs and their relationships. Using article characteristics such as authors, institutions, references (Price, 1963), and number of citations, it is classically used to evaluate the impact of scientific research, at different levels of organization, including articles, journals, researchers, or institutions. As such, bibliometrics reveals the habits of a research community. In a recent paper, Bedessem et al. (2021) compared publication rates of papers derived from the French national “Vigie Nature” CitSci program with that of other ecology papers and revealed that the yearly number of citations of articles using data from this CitSci program was higher than that of other articles in ecology. Despite the fact that citation rates may not be an accurate estimate of scientific relevance, this result suggests that CitSci might have a substantial epistemological impact on ecological research. The analysis of citation rates tells nothing of the research topics that are covered by CitSci. Another use of bibliometrics is gaining importance: thematic analysis. Based on a collection of keywords characterizing a research output, it allows the detection, quantification, and visualization of themes covered by a literature corpus (Cobo et al., 2011).

The present article addresses the specificity of ecological research relying upon citizen science. Focusing on ecological scientific articles published between January 2011 and December 2022, we used bibliometric tools to compare articles tagged as “citizen science” by their authors with that that did not use this term. We specifically asked the following questions: (i) what is the quantitative importance of CitSci in ecological academic literature? and which journals publish CitSci articles? (ii) what are research themes in ecology that are particularly likely to be addressed with CitSci, (iii) does CitSci rely on particular approaches (objects or methods) that differ from non‐participatory research? By doing so, this article establishes the current status of CitSci in ecological research and discusses opportunities for future developments of citizen science.

## 2 MATERIALS AND METHODS

### 2.1 Corpus preparation

We interrogated the Web of Science (WoS) database in February 2023, limiting our research to articles belonging to the WoS category ECOLOGY. We further limited our research to articles published between January 2011 and December 2022 because previous studies reported that citizen science bloomed in the scientific literature from 2010 on (Follett & Strezov, 2015; Kullenberg & Kasperowski, 2016). We used two independent requests. First, we searched for the strings {“Citizen science” OR “participatory science”} in all fields of the core WoS database that covers journals, books and book chapters and conference proceedings. This represented the “CitSci” corpus. Then, we repeated the search with the strings {NOT “Citizen science” NOT “participatory science”}, which represented the “non‐CitSci” corpus.

Filtering articles belonging to the WoS category ECOLOGY excludes multidisciplinary journals because WoS categories are defined at the journal level. Our search therefore excluded multidisciplinary journals such as *Nature*, *Nature Communications*, *Royal Society Open Science*, *Proceedings of the National Academy of Sciences of the United States of America*, *Science*, *Science Advances*, *Scientific Reports*, or the *PLoS Journals*. Yet, when we searched for CitSci articles in these journals, we noticed that several covered disciplines such as psychology, medicine, education science, or Earth science, in addition to ecology. Having considered these journals in our initial search would have generated an uncontrolled variability of keywords used in both the CitSci and non‐CitSci corpus. We therefore opted for a more conservative approach.

We extracted full records of the WoS database for papers published between 2011 and 2022 in both corpora. We only retained references corresponding to “articles,” that is, excluding reviews, editorials, corrections, conference abstracts, or book chapters. We excluded articles without abstract (2402 articles, 1%) or keywords (23,140, 10%). They corresponded to letters, responses, or opinion papers (Figure S1).

We manually checked the keywords used in the CitSci corpus to identify CitSci‐related keywords (specifically: citizen science, citizen science, participatory science, participatory research, citizen scientist, citizen scientists, volunteer, volunteers, participants, crowd sourcing, crowd‐sourcing, crowdsourcing, crowdsourced, contributors, community science, citizen sciences, citizen data, citizen datum, citizen science information system, citizen science information systems, citizen science data, citizen science datum, citizen initiative, citizen initiatives, citizen science data, citizen science datum, citizen sensors, citizen sensor, citizen‐reported data, citizen‐reported datum, citizen‐sourced data, citizen‐sourced datum, citizen monitors, citizen monitor, volunteer data, volunteer monitoring, amateur, amateurs, and amateur naturalists). We used authors' keywords rather than the “Keywords plus” variable provided by the WoS database as the latter is based on an algorithm extracting information from the reference list of an article and therefore does not reflect the authors' primary intention. Of the 1260 CitSci articles constituting the original CitSci corpus, 993 (i.e., 74%) used the abovementioned terms in abstracts. Because citizen science may not be referred as such in article keywords, we then searched for CitSci‐related keywords in abstracts of articles constituting the non‐CitSci corpus. By doing so, we added 1933 articles to the original CitSci corpus (Figure S1). The CitSci corpus therefore consisted of 3193 articles.

We deleted CitSci‐related keywords from the CitSci corpus to avoid having further analyses biased toward these keywords. Indeed, we expected that these terms would be among the most frequent in the CitSci corpus, which would have led us to conclude that the CitSci corpus is defined by CitSci‐related keywords. We switched plural terms to singular. We did not assign individual species names to a taxonomic group.

We used a two‐step approach to explore whether the CitSci corpus differs from the non‐CitSci corpus in terms of research themes or methods. We first compared the CitSci corpus with the non‐CitSci corpus, representative of ecology as a whole. The comparison may have been biased by the large difference in the number of articles in each corpus (3193 vs. 207,400). To avoid it, we used rarefaction, a classical approach in community ecology. It consists in randomly drawing in the largest sample a number of samples equivalent to that found in the smallest one (Gotelli & Colwell, 2001). There were 3193 articles in the CitSci corpus; we therefore drew 3193 articles at random in the non‐CitSci corpus (out of 207,400, see Figure S1).

Second, we narrowed the analysis by selecting in the complete non‐CitSci corpus only articles published in journal issues with at least one CitSci article (henceforth *CitSci publishing journals*, Figure S1). To ensure fair comparisons, we randomly drew *n* non‐CitSci articles in each issue of a CitSci publishing journal, where *n* is the number of CitSci articles in this issue (Figure S1). This second comparison is a way to control for likely differences among research areas in ecology. It thus brings complementary information. Assuming that CitSci publishing journals have their own scope, which may be narrower than ecology as a whole, comparing CitSci articles with non‐CitSci articles from CitSci publishing journals permits to analyze whether the participation of nonprofessional scientists is influential for the choice of research objects (e.g., habitats and taxa) or methods.

### 2.2 Statistical analyses

#### 2.2.1 Descriptive statistics

In order to provide an overview of publishing habits related to citizen science in the field of ecology, we calculated the number and proportion of CitSci articles among journals. For each corpus (CitSci, complete non‐CitSci corpus, non‐CitSci corpus from CitSci publishing journals), we counted the frequency of occurrence of each keyword and compared the most frequent keywords in each corpus.

#### 2.2.2 Thematic analysis

We first analyzed keyword co‐occurrence, representing associational strength between keywords (Figure S2). Specifically, the procedure follows three steps (Aria et al., 2022). First, a *p* × *q* presence‐absence matrix **D** is computed, whose elements *d*_*ij*_ represent the presence (1) or absence (0) of the *i*th keyword (among *q* keywords) in the *j*th article (among *p* articles). Because articles typically include more than one keyword, *q* is larger than *p*. **D** is then converted into a *q* × *q* matrix **A** whose generic elements *a*_*i,i′*_ (with *i* ≠ *i′*) represent the number of articles in which keywords *i* and *i′* co‐occur. The diagonal *a*_*i,i*_ represents the total number of articles in which the keyword *i* appears. **A** is converted in a second *q* × *q* matrix A* representing association strength (AS) between pairs of keywords such as AS_*i,i′*_ = *a*_*i,i′*_/(*â*_*i,i*_ × *â*_*i′,i′*_), where *â*_*i,i*_ and *â*_*i′,i′*_ are the expected number of occurrences of *i* and *i′* under the null hypothesis *H*_0_ that *i* and *i′* are statistically independent. AS_*i,i′*_ ranges from zero (*i* and *i′* do not co‐occur) and 1 (*i* and *i′* all co‐occur in the list of authors' keywords of every paper). It can also be referred to as equivalence index (Cobo et al., 2011) and is analogous to similarity indices that are classically used in community ecology.

We represented associational strength between keywords (A*) in a co‐occurrence network and used a *community detection algorithm* proposed by Blondel et al. (2008) to detect clusters of keywords strongly associated among each other. Their clustering algorithm (also referred to as Louvain algorithm) selects clusters that optimize network modularity, which is the difference between the number of edges (links) between vertices (keywords) within a given cluster and the expected number of edges in an equivalent network with edges placed at random. The Louvain algorithm was shown to provide a satisfying balance between accuracy and computing time (Lancichinetti & Fortunato, 2009; Yang et al., 2016). Here, clusters represent *research themes*. We ran the community detection algorithm 100 times, for the CitSci and non‐CitSci corpus. For each iteration, we extracted the vertices (i.e., keywords) characterizing each cluster.

**FIGURE 1:**
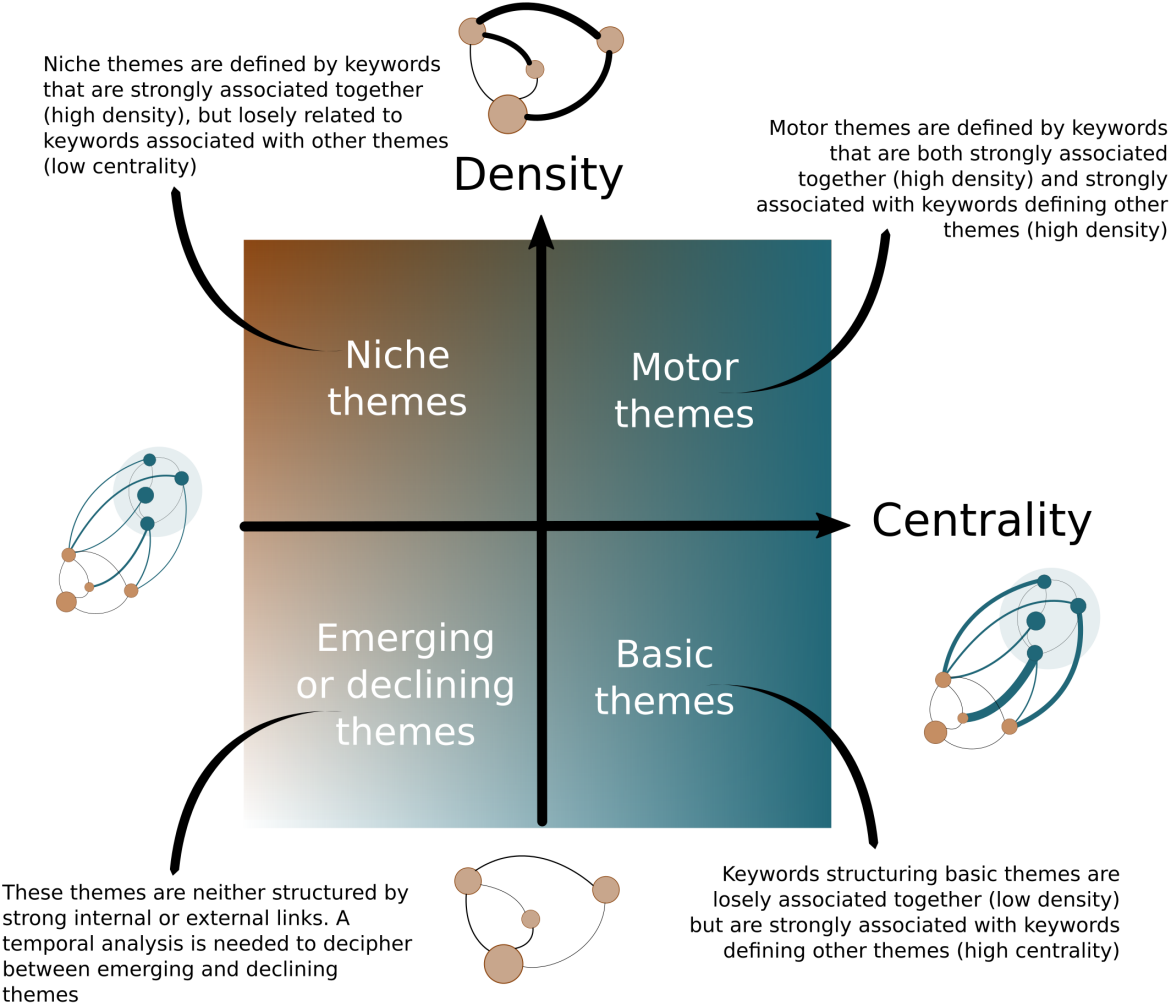
Schematic representation of keyword centrality and density defining basic, motor, niche, and emerging or declining themes in a thematic analysis. See main text for detailed description.

We then ran a *thematic analysis* to visualize research themes based on their centrality and density in a network analysis (Figure 1). A standard approach is to order keywords along two orthogonal axes (Cobo et al., 2011). The first axis is *centrality*. It is proportional to the sum of associational strength between keywords belonging to a given theme, and keywords belonging to another theme. Said differently, centrality represents the strength of external ties between a theme and other themes. The stronger keywords of a theme are connected to keywords of other themes, the more central the theme that these keywords define is, and the more important it is for the development of the discipline. The second axis is *density*. Contrarily to centrality, density reflects the strength of internal ties between keywords defining a theme. It represents how strongly keywords defining a theme are associated together and can be understood as a measure of the degree of maturity of a theme (Aria et al., 2022; Cobo et al., 2011). The density‐centrality biplot can be partitioned into four quadrants (Figure 1) representing niche (high density, low centrality), motor (high density, high centrality), basic (low density, high centrality), and emerging/declining (low centrality, low density) themes (Aria et al., 2022). We repeated the thematic analysis 100 times and plotted the centrality and density of each cluster, averaged across the 100 repetitions.

We produced keyword co‐occurrence networks and thematic analyses for CitSci and non‐CitSci articles separately using package bibliometrix (Aria & Cuccurullo, 2017) in R environment (R Core Team, 2022), and in particular functions *biblioNetwork*, *networkPlot*, and *thematicMap*. We performed the analysis of the non‐CitSci corpus twice, first with the subset of articles randomly drawn from ecological literature as a whole, and second with the subset of non‐CitSci articles randomly drawn from CitSci publishing journals.

## 3 RESULTS

**FIGURE 2:**
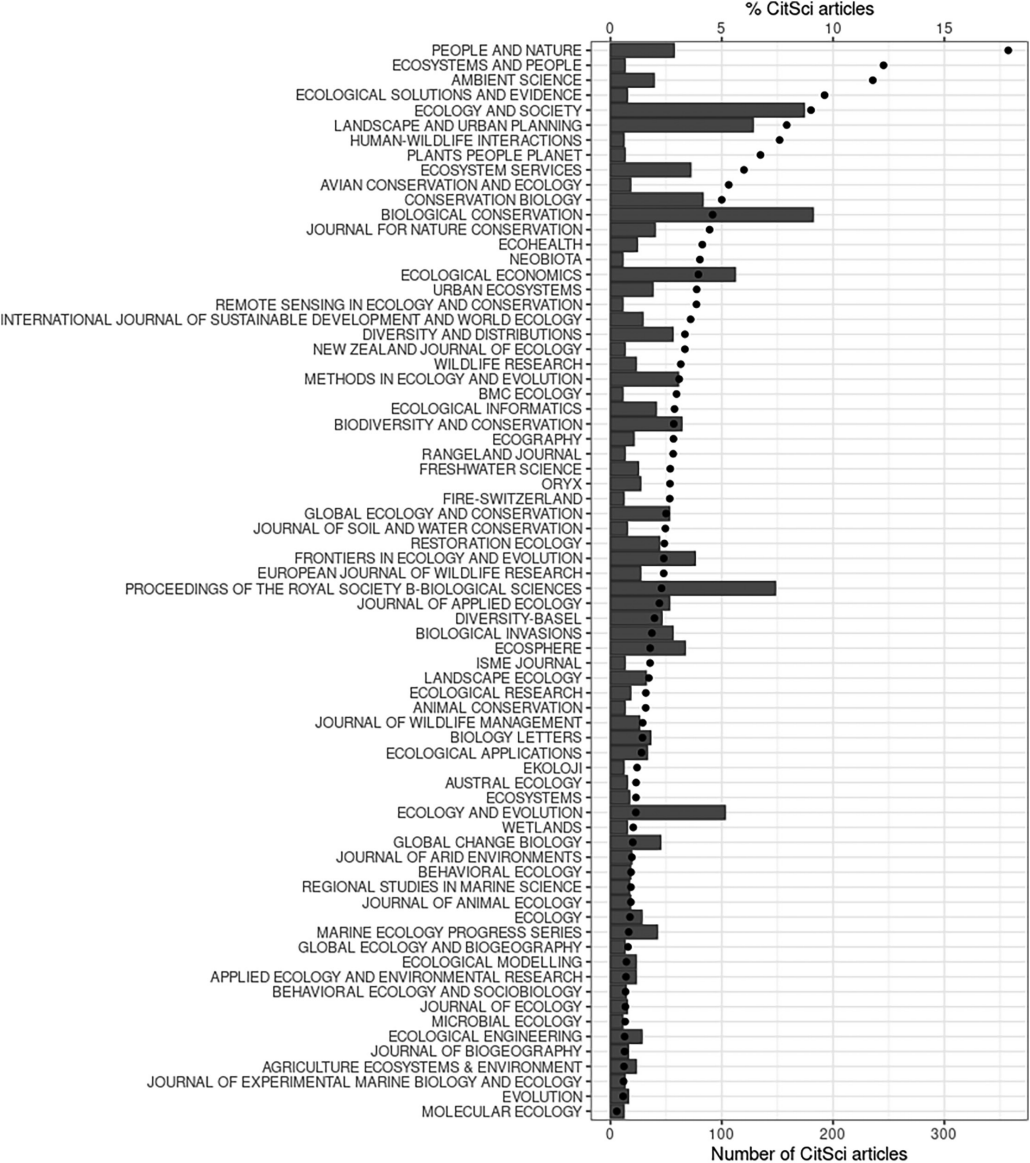
Percentage (dots) and number (bars) of CitSci articles published in CitSci publishing journals between 2011 and 2022. Journals are ordered according to the percentage of CitSci articles they published. Journal names are those returned by the Web of Science database. For the sake of clarity, of the 134 journals having published at least one CitSci article over the period, we only show those having published at least 10 CitSci articles (72 journals).

We primarily analyzed the content of 210,593 articles published in 14,338 issues of 209 journals between 2011 and 2022. Scientific articles relying on citizen science (CitSci articles) in the field of ecology only represented a small proportion of articles published in the field (<1%), but this proportion increased regularly between 2011 and 2022 (Figure S3). Of the 209 journals constituting the corpus, 134 published one CitSci article or more (i.e., 64%), with a great variability in the number and proportion of CitSci articles (Figure 2). The number of CitSci articles per journal issue varied between 1 and 25 (average: 1.46), which corresponds to 0.5% and 72.2% (mean: 8.4%, median: 6.7%) of articles published in the corresponding issues. The skewness of the distribution resulted from the presence of some special issues on citizen sciences (e.g., *Freshwater science*, Vol. 38, Number 2, June 2019; *Conservation Biology*, Vol. 30, Number 3, 2016).

**FIGURE 3:**
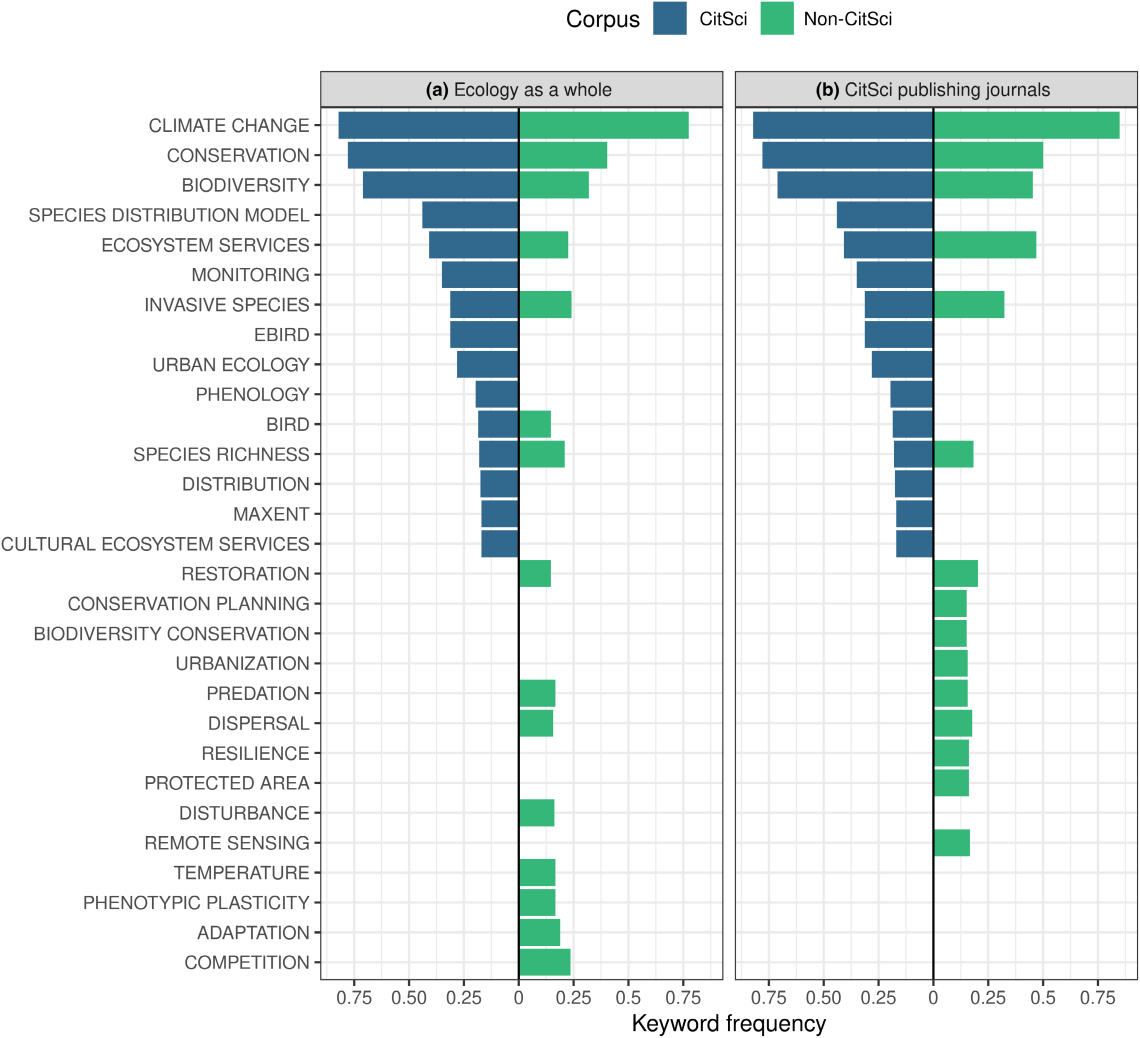
Frequency of keywords used in the CitSci (blue) and non‐CitSci (green) corpora. Only the 15 most frequent keywords of each corpus are shown. (a) Keyword frequency in the non‐CitSci corpus was assembled from articles in the field of ecology as a whole. (b) Keyword frequency in the non‐CitSci corpus was assembled from CitSci publishing journals. The absence of blue or green bars on the left‐ and right‐hand sides indicates that corresponding keywords were only present in the CitSci or non‐CitSci corpora, respectively.

“Climate change,” “biodiversity,” and “conservation” were the most common keywords used in the CitSci corpus and the non‐CitSci, regardless of whether the non‐CitSci corpus consisted of articles in ecology as a whole or of articles published in CitSci publishing journals (Figure 3). Keywords such as “invasive species” or “ecosystem services” were also shared by both corpora, but were more frequent in the CitSci corpus and in the non‐CitSci corpus from CitSci publishing journals (Figure 3). In contrast, “species distribution model,” “urban ecology” (and “urbanization”), and “ebird” (a citizen science program) were only present in the CitSci corpus, whereas “disturbance,” “predation,” “dispersal,” or “functional traits” were only present in the non‐CitSci corpus (Figure 3).

**FIGURE 4:**
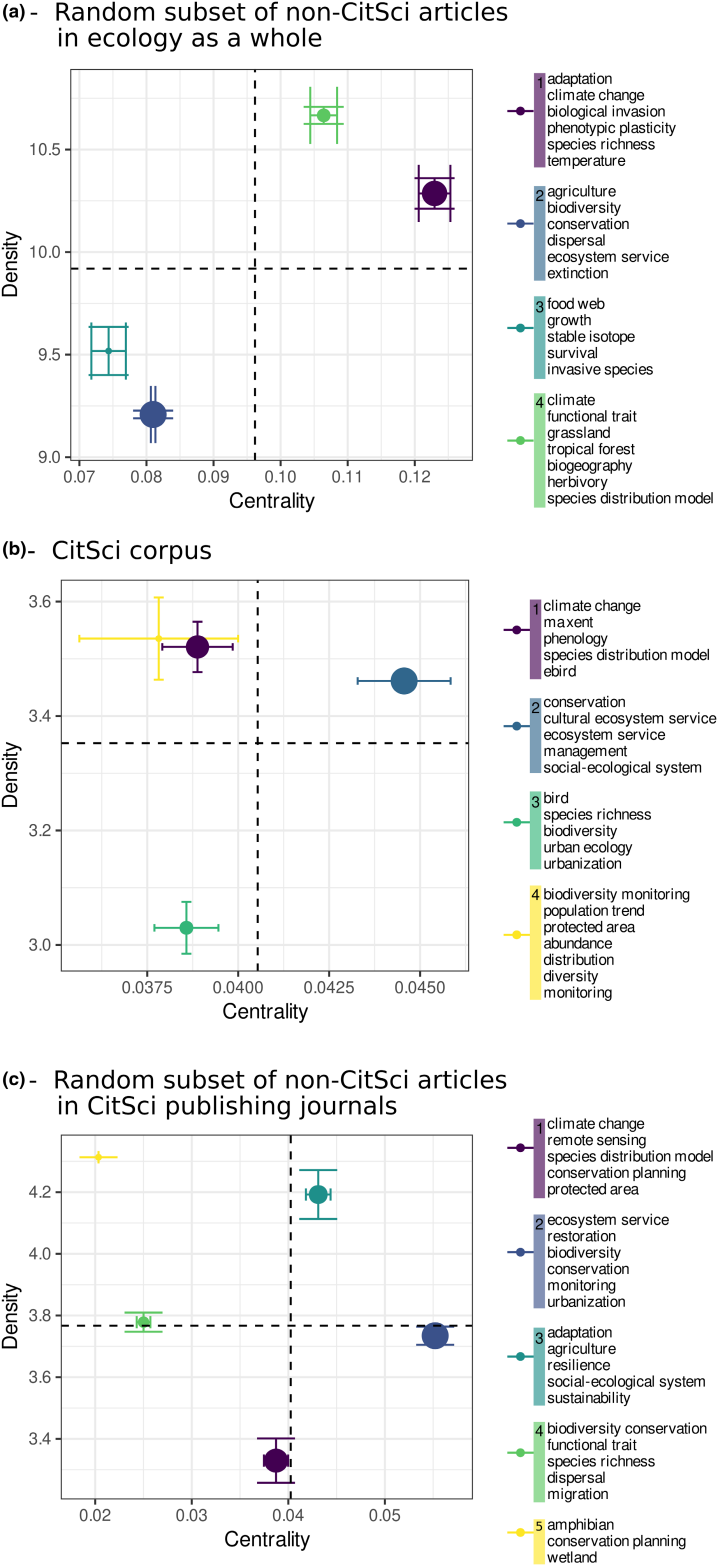
Thematic analysis of keywords describing CitSci and non‐CitSci articles. Dots represent research themes defined as the combination of mean (±SD) centrality and mean density of clusters of keywords calculated across 100 association networks. The central panel (b) corresponds to the CitSci corpus. The upper (a) and lower (c) panels correspond to the non‐CitSci corpora assembled from articles in the field of ecology as a whole (a) or articles from CitSci publishing journals (c).

Keyword co‐occurrence networks and thematic maps revealed more detailed patterns (Figure 4). Climate change and its consequences on species distribution and adaptation was a motor theme (high centrality and high density) in the non‐CitSci corpus assembled from a random subset of ecological literature (Cluster 1 in Figure 4a). This theme appeared as less central when we only considered non‐CitSci articles published in CitSci publishing journals (Cluster 1 in Figure 4c) and even less central in the CitSci corpus where it was identified as a niche theme (Figure 4b). The keyword “climate change” was also associated with different keywords, with similar high density, when comparing the CitSci corpus with the non‐CitSci corpus assembled from a random subset of articles in ecology. This finding suggests that climate change is considered through a different perspective when studied by ecologists only, or with the contribution of nonprofessional ecologists.

Conservation biology and its links with socio‐ecosystems and ecosystem services was a motor theme in the CitSci corpus (Cluster 2 in Figure 4b). This theme was also central, albeit less dense in the non‐CitSci corpus assembled from CitSci publishing journals (Figure 4c) but it appeared as much less a central theme in the non‐CitSci corpus assembled from the random subset of ecology articles (Cluster 4 in Figure 4a). These differences may reflect editorial specialization of journals that are more prone to publish CitSci articles. The status of biodiversity and related keywords also differed between the CitSci and non‐CitSci corpora. In the CitSci corpus, biodiversity was an important keyword associated with two themes (Figure 4c,d), an emerging theme addressing biodiversity in urban environments (Cluster 3), and a niche theme mostly focusing on the monitoring of biodiversity with citizen science (Cluster 4). In the non‐CitSci corpora, biodiversity was embedded in a cluster of keywords related to ecosystem services and restoration ecology that was identified as an emerging theme (Cluster 2 in Figure 4a,b) in ecology as a whole and as a basic theme in the non‐CitSci articles published in CitSci publishing journals. Finally, only in the non‐CitSci corpus emerged a theme related to functional ecology with the keyword “functional traits.”

## 4 DISCUSSION

We aimed to assess the relative importance of CitSci in ecological academic literature to determine whether some research areas in ecology are particularly likely to be addressed through CitSci. Our bibliometric analysis of articles published over the last decade revealed the differences and similarities between citizen science and classical ecology research, both regarding research themes and approaches (objects or methods). Specifically, we found that citizen science is well integrated in the main, classical research themes of ecology (biodiversity, conservation, and climate change), with two notable differences. First, our results suggest that CitSci articles are generally more descriptive regarding these research themes, whereas non‐CitSci research is more explanatory (i.e., focused on mechanisms). Second, these themes do not have the same status in the CitSci and non‐CitSci corpora: Climate change and its consequences on species distribution and adaptation is a motor theme in the non‐CitSci corpus, whereas the motor theme of the CitSci corpus is conservation biology and its implications on socio‐ecosystems and ecosystem services.

### 4.1 CitSci research in ecology is more descriptive than explanatory

A first finding is that non‐CitSci research appears to be essentially oriented toward the search for mechanisms driving biodiversity patterns in response to climate change and anthropogenic pressure (explanatory research), whereas CitSci research is more descriptive. Indeed, even if biodiversity and climate change are the most frequent keywords in all corpora, they were not associated with the same sets of keywords: only in the non‐CitSci corpus appeared keywords referring to evolutionary processes (“adaptation” and “phenotypic plasticity”), biotic interactions (“dispersal,” “herbivory,” and “food web”) and functional ecology (“functional traits”). By contrast, CitSci seems to consist in the collection of a great number of biodiversity data to describe biodiversity (“biodiversity monitoring” and “species distribution model”).

A possible explanation for this difference is that there is a negative selection against CitSCi in explanatory research. The study of mechanisms driving patterns generally requires experimental and instrumental approaches that are not accessible (physically or financially) to the general public (Pocock et al., 2014), whereas sharing observational data is much easier. More generally, fundamental questions in ecology may be less appealing to the general public than the monitoring of emblematic species, thus imposing a recruitment constraint determining which type of research can be conducted with the public (Larson et al., 2020; Pocock et al., 2018). Alternatively, there might be a positive selection in favor of CitSCi when it comes to monitor biodiversity across time and space, because professional scientists can hardly address these questions alone with—often—limited resources.

We note that descriptive research does not imply that citizen science‐based data collection could not give rise to important explanatory findings. Notably, one could expect that citizen science programs might ground data‐driven science, in the sense of Leonelli (2016), such as the identification of regularities that point toward the generation of hypotheses regarding the mechanisms determining biodiversity dynamics (Calba et al., 2014; Ekholm et al., 2019; Julliard et al., 2006).

### 4.2 Biodiversity is a central theme in citizen science research, whereas climate change is emerging

Overall, it is worth noting that the thematic content of the CitSci corpus was more comparable with that of the non‐CitSci corpus when the latter was assembled from articles published in CitSci publishing journals than when it concerned ecology as a whole. These observations may reflect the editorial specialization of journals, some being more likely to publish CitSci articles than others. That said, an important finding regarding research themes is that climate change and biodiversity have different status in the CitSci and non‐CitSci corpora.

Climate change and its consequences on species distribution and adaptation is a motor theme in the non‐CitSci corpus whereas biodiversity, biological conservation and their relationship to ecosystem services is an emerging theme in this corpus. In other words, most of ecological research of the last decade was related to climate change, regardless of the level of organization (individuals, populations, species, communities) or habitat, whereas biodiversity studies were less connected with other subfields of ecology. This difference may be representative of the time lag between the bloom of climate change studies and the more recent focus on its impact on biodiversity (Anderson et al., 2021; Legagneux et al., 2018).

On the contrary, research on climate change constitutes a niche theme in the CitSci corpus, with a focus on phenology and methods for the analysis of opportunistic data collected by citizen scientists. The motor theme of the CitSci corpus is conservation biology and its implications on socio‐ecosystems and ecosystem services. This association between conservation and its human dimensions suggests that CitSci tends to develop social and economical perspectives on conservation. Since it depends on the motivations of citizens to participate in research, CitSci might then give a specific importance to environmental issues that affect them directly. This finding is interesting, since one of the promises of CitSci is to guide research toward more socially relevant topics and/or to take into account the consequences of scientific research for human lives (Turrini et al., 2018). Another interesting finding is the presence of an emerging theme within the CitSCi corpus that can be referred to as “biodiversity in the city” (Cluster 3 in Figure 4c,d). This may reflect the fact that most of the human population now lives in cities; consequently, there is a large pool of urban participants to citizen science projects (Roger & Motion, 2022).

### 4.3 Limitations and future directions

We acknowledge a possible discrepancy between our dataset (published papers) and the reality of citizen science practice in ecology. First, not every article using data collected by volunteers mentions citizen science in their abstract or keywords (Cooper et al., 2014). Second, our analysis of the thematic content of the CitSci literature is limited to articles published in indexed journals. Yet, there is a time lag between the onset of a research project and the publication of the results in scientific journals. This lag may be wider in the case of CitSci programs as they require the building of a network of volunteers before a sufficient amount of reliable data is collected. It would then be interesting to complete our bibliometric analysis with an examination of the current citizen science projects that are being conducted, in order to assess their goals and their expected scientific impacts. In addition, a large proportion of projects referred to as citizen science projects do not primarily aim at the publication of scientific articles in indexed journals, because they use other forms of dissemination of the results, or because they address issues that are mostly relevant at a very local scale (Kullenberg & Kasperowski, 2016). Strictly speaking, our method offers a description of citizen science as it is diffused under the form of scientific publications. This may not reflect the diversity of citizen science initiatives. Qualitative approaches will be needed to complement our assessment.

## 5 CONCLUSION

The production of ecological knowledge over the last decade greatly benefited from the participation of nonprofessional scientists in the acquisition and analysis of ecological data. Some aspects of ecological sciences were particularly prone to be studied through citizen science, while others have remained the prerogative of professional scientists.

Broadly, the same research themes were to be found in the CitSci and non‐CitSci corpora, with only subtle differences in the relative importance of particular themes and in the way these themes are tackled (in terms of research methods and objects). Merely, CitSci and non‐CitSci research differed in the objects that are studied (urban ecosystems easily accessible to citizen scientists vs. tropical forests and grasslands mostly accessible to professionals), the status of climate change and biodiversity research in the whole ecology field, and the relative importance of explanatory research.

With a strict focus on the last decade, our results complement previous scientometric approaches supporting CitSci as a legitimate method to produce ecological knowledge (Bedessem et al., 2021; Pelacho et al., 2021). Looking forward, it is sensible to speculate that the development of methodological (statistical) tools for the analysis of citizen science data—currently a niche theme—prefigures a shift toward the development of more explanatory approaches and the attenuation of epistemic differences between CitSci and non‐CitSci research.

## AUTHOR CONTRIBUTIONS

**Bastien Castagneyrol:** Conceptualization (equal); formal analysis (lead); methodology (lead); visualization (lead); writing – original draft (equal); writing – review and editing (equal). **Baptiste Bedessem:** Conceptualization (equal); methodology (supporting); writing – original draft (equal); writing – review and editing (equal). **Romain Julliard:** Conceptualization (equal); methodology (supporting); writing – original draft (supporting); writing – review and editing (equal).

## CONFLICT OF INTEREST STATEMENT

None.

## Supporting information


Figures S1–S3

## Data Availability

The data and codes used in the present study are archived on the institutional repository www.entrepot.recherche.data.gouv.fr (Castagneyrol et al., 2022, https://doi.org/10.57745/CSQCIC).
