# Supplementary material for: Is ecology different when studied with citizen scientists? A bibliometric analysis
Source: Ecol Evol. 2023 Sep 19;13(9):e10488. doi: 10.1002/ece3.10488 (PMC10509151; doi:10.1002/ece3.10488)
Supplement: Supplementary file 1 — Figures S1–S3 [file ECE3-13-e10488-s001.docx]

# Supplementary figures


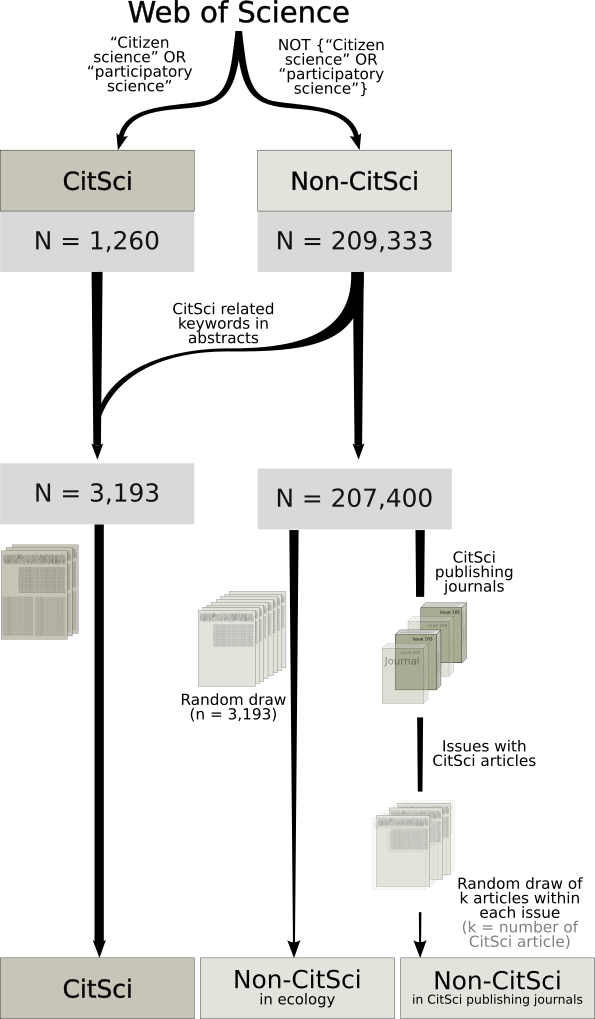


**Figure S1.** Schematic representation of the construction of CitSci and non-CitSci corpora from two parallel queries in the Web of Science database.


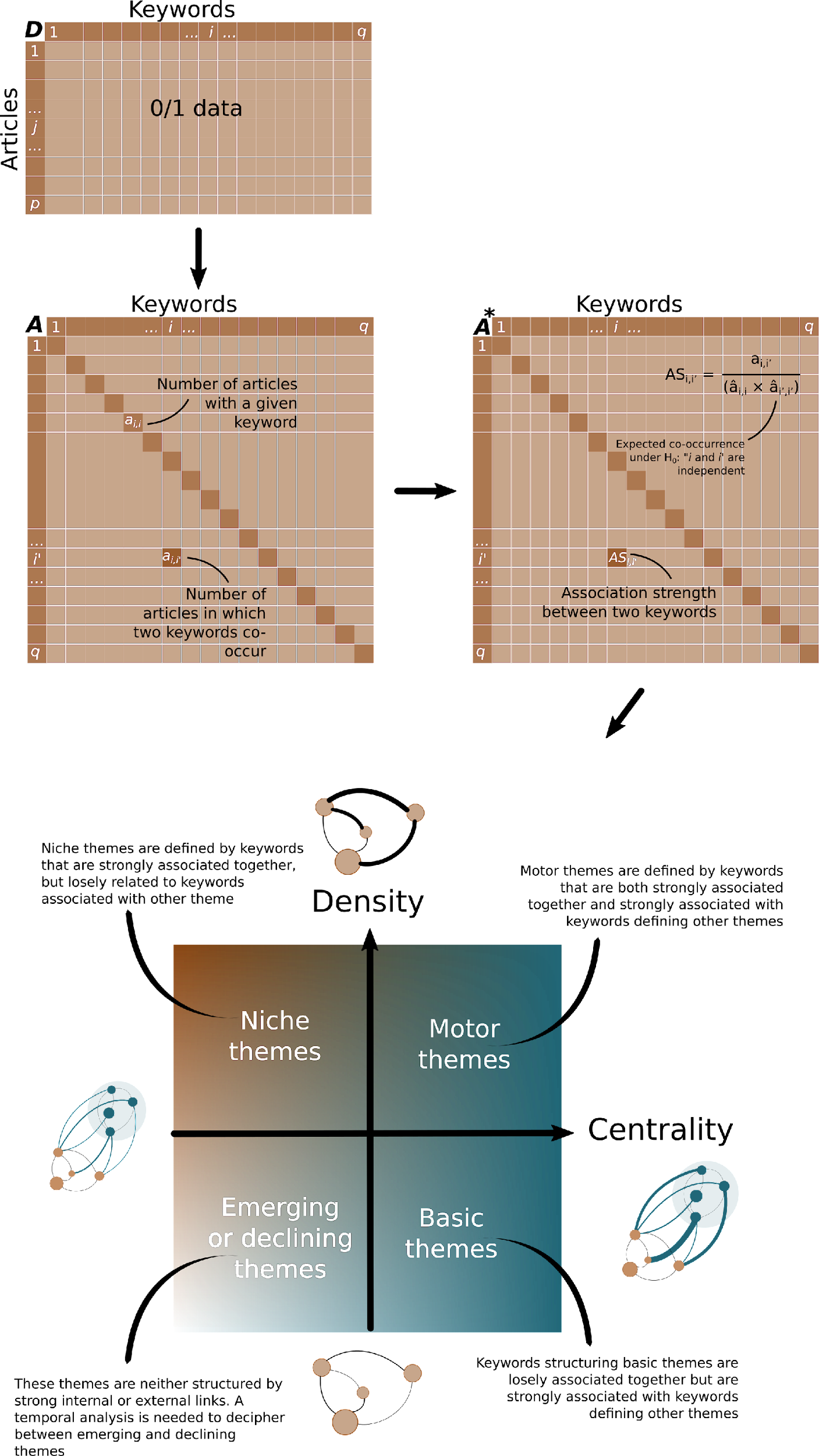


Figure S2. Schematic representation of the construction of the thematic analysis. Letters at the top left corner of each matrix correspond to its name; see main text for details.


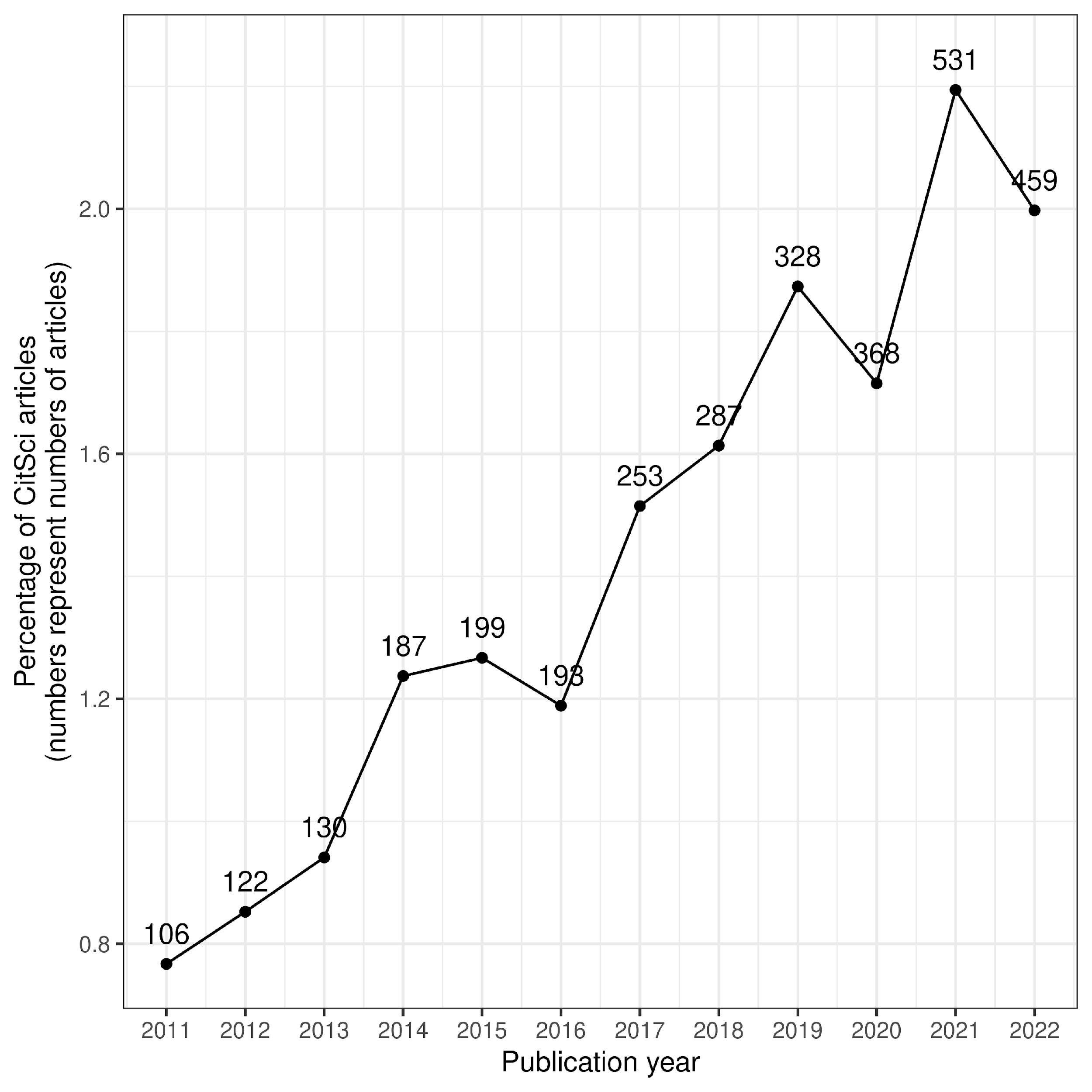


**Figure S3. Dynamics of CitSci publishing between 2011 and 2022.** Dots represent the percentage of CitSci articles in the field of ecology (100 × number of CitSci articles / (Number of CitSci articles + Number on non-CitSci articles)). Numbers above dots represent the number of articles.
